# Clinical and electroencephalogram characteristics of methylmalonic acidemia with MMACHC and MUT gene mutations

**DOI:** 10.1186/s12887-024-04559-8

**Published:** 2024-02-14

**Authors:** Yujun Yuan, Ying Ma, Qiong Wu, Liang Huo, Chun-Feng Liu, Xueyan Liu

**Affiliations:** 1 Department of Pediatrics, Shengjing Hospital of China Medical University, Shenyang, China; 2 Department of Neurology, Shengjing Hospital of China Medical University, Shenyang, China

**Keywords:** Methylmalonic academia, Epilepsy, MRI, Developmental delay, EEG, Epilepsy

## Abstract

**Objective:**

This study investigated the clinical, imaging, and electroencephalogram (EEG) characteristics of methylmalonic acidemia (MMA) with nervous system damage as the primary manifestation.

**Methods:**

From January 2017 to November 2022, patients with nervous system injury as the main clinical manifestation, diagnosed with methylmalonic acidemia by metabolic and genetic testing, were enrolled and analyzed. Their clinical, imaging, and electroencephalogram data were analyzed.

**Results:**

A total of 18 patients were enrolled, including 15 males and 3 females. The clinical symptoms were convulsions, poor feeding, growth retardation, disorder of consciousness, developmental delay, hypotonia, and blood system changes. There were 6 cases (33%) of hydrocephalus, 9 (50%) of extracerebral space widened, 5 (27%) of corpus callosum thinning, 3 (17%) of ventricular dilation, 3 (17%) of abnormal signals in the brain parenchyma (frontal lobe, basal ganglia region, and brain stem), and 3 (17%) of abnormal signals in the lateral paraventricular. In addition, there were 3 cases (17%) of cerebral white matter atrophy and 1 (5%) of cytotoxic edema in the basal ganglia and cerebral peduncle. EEG data displayed 2 cases (11%) of hypsarrhythmia, 3 (17%) of voltage reduction, 12(67%) of abnormal discharge, 13 (72%) of abnormal sleep physiological waves or abnormal sleep structure, 1 (5%) of immature (delayed) EEG development, and 8 (44%) of slow background. There were 2 cases (11%) of spasms, 1 (5%) of atonic seizures, and 1 (5%) of myoclonic seizures. There were 16 patients (89%) with hyperhomocysteinemia. During follow-up, 1 patient was lost to follow-up, and 1 died. In total, 87.5% (14/16) of the children had varying developmental delays. EEG was re-examined in 11 cases, of which 8 were normal, and 3 were abnormal. Treatments included intramuscular injections of vitamin B12, L-carnitine, betaine, folic acid, and oral antiepileptic therapy. Acute treatment included anti-infective, blood transfusion, fluid replacement, and correcting acidosis. The other treatments included low-protein diets and special formula milk powder.

**Conclusion:**

Methylmalonic acidemia can affect the central nervous system, leading to structural changes or abnormal signals on brain MRI. Metabolic screening and genetic testing help clarify the diagnosis. EEG can reflect changes in brain waves during the acute phase.

## Introduction

Methylmalonic acidemia is an autosomal recessive metabolic disorder caused by a deficiency in the methylmalonic acid CoA mutant enzyme or the enzyme metabolizing cobalamin (vitamin B12). In the body, four amino acids (isoleucine, methionine, threonine, and valine), odd-chain fatty acids, and cholesterol can produce propionyl CoA, which is then converted into methyl malonyl CoA and then transported into the mitochondria for the tricarboxylic acid cycle via an enzymatic reaction. The conversion of propionyl CoA to methyl malonyl CoA requires the participation of enzymes, the most important of which is methyl malonyl CoA mutase and its coenzyme 5'-deoxyadenosine cobalamin (AdoCbl, which is derived from vitamin B12 through a series of enzymatic reactions). Accordingly, either an innate genetic abnormality in the methyl malonyl CoA mutase or a disorder in any part of the evolution of vitamin B12 into an AdoCbl can lead to the accumulation of methyl malonyl CoA and lead to methylmalonic acidemia. In addition, another coenzyme of methylcobalamin (MeCbI) which is derived from vitamin B12 is necessary to convert homocysteine to methionine, a process that can lead to hyperhomocysteinemia if impaired [1, 2].

Depending on the presence of homocysteinemia, MMA can be divided into isolated MMA or methylmalonic acidemia with homocystinuria [3]. Isolated MMA includes *mut*^*–*^ and *mut*^*0*^ types caused by mutations in the mut gene, cblA type caused by mutations in the MMAA gene, cblB type caused by mutations in the MMAB gene, and Tcblr type caused by mutations in the CD320 gene. Homocysteinemia associated with MMA includes cblC, cblD, cblF, cblJ, and cblX types. Among them, cblC (also known as cobalamin C disease or cblC disease) is the most common inherited disorder of cobalamin (vitamin B12) metabolism, caused by the MMACHC mutation located on chromosome 1p34.1. cblD, cblF, cblX, and cblJ are caused by mutations in the MMADHC gene located on chromosome 2q23, LMBRD1 gene located on chromosome 6q13, HCFC1 gene located on chromosome Xq28, and ABCD4 gene located on chromosome 14q24, respectively [4, 5].

The onset period of MMA includes anorexia, lethargy, hypotonia, progressive renal failure, functional immune impairment, hematological abnormalities, and other multisystem injuries [4]. Among them, the damage to the nervous system is the most severe and has a high disability rate [6]. Central nervous system manifestations of methylmalonic acidemia in the early stage include microcephaly, seizures, psychomotor delay, lethargy, feeding difficulties, hypotonia, etc. [7]. According to the age of onset, cb1C can be divided into early onset (onset within one year) and late-onset (after four years), with different clinical manifestations according to the type and location of gene mutations [8]. Early onset is associated with a higher mortality risk [9]. Later onset patients or adults may display cognitive abnormalities such as progressive encephalopathy, speech disorders, learning difficulties, psychoneurotic symptoms, dementia, and executive dysfunction and movement disorders. It can also manifest as spinal cord degeneration and thrombosis [9, 10]. In addition, it can affect the optic nerve [11], resulting in impaired vision.

Common brain MRI signs of MMA include dysmyelination, brain atrophy, lateral ventricle dilatation, and bilateral symmetric pallidum signal abnormalities [6]. EEG can reflect changes during the acute and convalescent periods. The first or main symptoms of MMA can be mainly concentrated in the nervous system; therefore, brain MRI and EEG can reflect the brain structural changes and electrophysiological changes of MMA patients, respectively.

## Methods and materials

### Patients

MMA patients with nervous system damage symptoms who were admitted to the Department of Pediatric Neurology, Shengjing Hospital of China Medical University, from January 2017 to November 2022 were reviewed. In addition, general information, clinical manifestations, genetic testing (Table 1), laboratory examination (Table 2), brain MRI and EEG (Table 3), and prognosis data of the patients (Table 3) were collected.

**Table 1:** General data and genetic testing results of 20 patients

	**N**	**Age of onset**	**Gender**	**Main symptoms on admission**	**Mutant gene**	**Site of mutation**
**MMACHC gene mutation**	1	59 d	Male	Poor feeding, lethargy, developmental delay	Compound heterozygous mutations in MMACHC, Type CblC	c.315(exon 3) C > G; c.609(exon 4) G > A
	2	2 m	Female	Poor feeding, lethargy, developmental delay	Compound heterozygous mutations in MMACHC, Type CblC	c.567(exon)c.568(exon4)ins T; c.609(exon 4) G > A
	3	3 m	Male	Developmental delay, motor developmental abnormality, Lack of eye contact	Compound heterozygous mutations in MMACHC, Type CblC	c.217(exon2) C > T; c.656(exon4)c.658(exon4) del AGA
	4	2 y1 m	Male	Decreased autonomic activity, slow response and lethargy. motor and language developmental delay	Compound heterozygous mutations in MMACHC, Type CblC	c.615(exon4)C > A; c.394(exon 3)C > T
	5	2 m	Male	Developmental delay, motor developmental abnormality, intermittent quadriplegia	Compound heterozygous mutations in MMACHC, Type CblC	c.609( exon4)G > A; c.658(exon4)_660(exon4)del
	6	4 y	Male	Developmental delay, intellectual disability, language developmental delay, motor developmental abnormality	Compound heterozygous mutations in MMACHC, Type CblC	c.609(exon 4)G > A; c.566(exon 4)_c.567(exon 4)ins T
	7	4 m	Male	Eyes looking down, drowsiness, lower limb weakness, convulsions, developmental delay	One homozygous mutation in MMACHC, Type CblC	c.609(exon 4) G > A
	8	4 m	Male	Developmental delay, motor developmental abnormality, spasm seizures, atonic seizures	Compound heterozygous mutations in MMACHC, Type CblC	c.217(exon2) C > T; c.656(exon4)_c.658(exon4)del AGA
	9	1 y6 m	Male	Convulsions, lethargy, developmental delay, language developmental delay, intellectual disability and motor developmental abnormality	Compound heterozygous mutations in MMACHC, Type CblC	c.365(exon 3) A > T; c.566(exon 4)_c.567(exon4) ins T
	10	5 m	Male	Convulsions, developmental delays, motor developmental abnormality	Compound heterozygous mutations in MMACHC, Type CblC	c.656(exon4)_c.658(exon4)del AGA; c.609(exon 4) G > A
	11	2 m	Female	Convulsions, developmental delay, growth retardation, dystonia	Compound heterozygous mutations in MMACHC, Type CblC	c.394(exon 3)C > T; c.656(exon4)_c.658(exon4) del AGA
	12	2 m	Male	Convulsions, developmental delay, growth retardation, poor feeding	Compound heterozygous mutations in MMACHC, Type CblC	c.217(exon2)C > T; c.609(exon4) G > A
	13	4 y	Male	Left limb weakness, developmental delay, motor developmental abnormality, lethargy	Compound heterozygous mutations in MMACHC, Type CblC	c.80(exon 1)A > G; c.609(exon 4) G > A
	14	5 m	Male	Convulsions, lethargy, developmental delay, growth retardation, motor developmental abnormality	Compound heterozygous mutations in MMACHC, Type CblC	c.656(exon4)_c.658(exon4)del AGA; c.609(exon4) G > A
	15	5 y	Male	Lethargy, convulsions, developmental delay, intellectual disability and motor developmental abnormality	Compound heterozygous mutations in MMACHC, Type CblC	c.609(exon4) G > A; c.658(exon4)_660(exon4) del
	16	5 m	Female	Convulsions, developmental delay, growth retardation, poor feeding	One homozygous mutation in MMACHC, Type CblC	c.609(exon4) G > A
**MUT mutation**	17	6 y	Male	Poor feeding, vomiting, lethargy, shortness of breath, metabolic acidosis, deep venous thrombosis of the lower extremity	Compound heterozygous of the MUT gene	c.1295(exon 6)A > C; c.1141(exon 6) G > A
	18	3 m	Male	Poor feeding	Compound heterozygous of the MUT gene	c.729(exon3)_c.730(exon3)ins TT; (exon:13) del

*N* Number, *y* year, *m* month

**Table 2:** Laboratory testing results

Group	Number	Age of onset	Gender	HCY	Blood system	Ammonia	Lactic acid	ALT	AST	Creatinine	Propionyl carnitine (C3)	Acetyl carnitine(C2)	C3/C2	Methylmalonic acid -2
MMA + HCY	1	59 d	Male	155↑	Anemia	9.4↓	2.25↑	41↑	44↑	31.3 N	6.71↑	9.21 N	0.73↑	105.1↑
	2	2 m	Female	> 198.4↑	Normal	8.7↓	3.15↑	72↑	49↑	29.1 N	7.95↑	104.84↑	0.08 N	46.5↑
	3	3 m	Male	195.26↑	Anemia	38.2↑	4.4↑	17.75 N	29.09 N	-	4.83 N	4.08↓	1.18↑	132.8↑
	4	2 y1 m	Male	157.17↑	Normal	23.7 N	1.5 N	41↑	48↑	30.4 N	3.1 N	5.79 N	0.54↑	86.3↑
	5	2 m	Male	147.82↑	Normal	78.4↑	6.1↑	2 N	24 N	18.4 N	6.64↑	11 N	0.6↑	56.3↑
	6	4 y	Male	113.6↑	Normal	15.9 N	3.14↑	15 N	31 N	31.2 N	15.01 ↑	17.23 N	0.87↑	246.6↑
	7	4 m	Male	203.2↑	RBC, WBC, and PLT decrease	20.3 N	2.4↑	10 N	15 N	21.1 N	5.2↑	4.17↓	1.25↑	56↑
	8	4 m	Male	217↑	Normal	87↑	4.6 ↑	19.1 N	17 N	24 N	6.4↑	27.63 N	0.23↑	-
	9	1 y6 m	Male	103.38↑	Normal	10.7↑	1.36↑	10 N	29 N	30.4 N	-	-	-	-
	10	5 m	Male	102.49↑	Normal	-	-	33 N	36↑	14.4 N	10.55↑	26.25 N	0.4↑	62↑
	11	2 m	Female	169.1↑	Normal	55.9↑	5.01↑	28 N	26 N	17.3 N	9.42↑	9.78 N	0.92↑	90↑
	12	2 m	Male	217.7↑	Normal	29.6 N	2 N	36 N	32 N	22.8 N	9.15↑	11.03 N	0.83↑	70↑
	13	4 y	Male	148↑	Normal	-	-	7 N	28 N	27 N	8.6↑	29.29 N	0.29↑	21.9↑
	14	5 m	Male	420↑	Normal	54.5↑	2.3↑	55↑	36↑	20.8 N	-	-	-	-
	15	5 y	Male	185.47↑	Normal	17.8 N	1.9 N	9 N	14 N	81.2↑	9.81↑	6.28 N	1.56↑	164.8↑
	16	5 m	Female	146↑	Normal	23 N	-	28N	24 N	24N	5.2 ↑	9.16N	0.56↑	60↑
Isolated MMA	17	6 y	Male	9.78 N	Normal	40.7↑	1.4 N	13 N	19 N	25.2 N	-	-	-	163↑
	18	3 m	Male	5.84 N	Normal	32 N	3↑	28 N	45 N	16 N	17.52↑	24.44 N	0.72↑	133.6↑

Homocysteine (HCY) normal range: 0–15 µmol/L; blood ammonia normal range: 11–32 µmol/L; blood lactic acid normal range: 0.7–2.1 µmol/L; alanine aminotransferase (ALT) normal range: 0–40 U/L; aspartate aminotransferase (AST) normal range: 5–34 U/L; creatinine normal range: 19–44 μmol/L; propionyl carnitine (C3) normal range: 0.5–5.0; acetylcarnitine (C2) normal range: 4.5–65; C3/C2 normal range: 0.02–0.20; methylmalonic acid-2 normal range: 0.0–4.0. RBC, red blood cell; WBC, white blood cell; PLT, platelet. “-”: unknown, missing data, or no corresponding test. “y”: year; “m”: month. “↓”: low, “N”: normal, “↑”: high

**Table 3:** MRI, EEG, and follow-up information

Group	N	Head MRI	Background of EEG	Sleep physiological wave/sleep cycle of EEG	Abnormal discharge, abnormal waveform and Seizure type during EEG monitoring	Age at follow-up	EEG at follow-up	The status of development at follow-up
**MMA + HCY**	1	Severe hydrocephalus	Generalized bihemispheric low voltage without physiological waves	Absence	Multifocal (sharp) slow waves appeared asynchronously, especially in the anterior	1y1m	Normal	Developmental language delay, intellectual disability, motor developmental abnormality
	2	Hydrocephalus	Generalized bihemispheric low voltage without physiological waves	Absence	Bilateral multifocal sharp wave, sharp slow wave, irregular θ wave activity was not synchronized, and irregular	1y1m	-	Developmental language delay, intellectual disability, motor developmental abnormality
	3	Hydrocephalus and cerebral parenchymal atrophy	4-5 Hz medium-amplitude θ wave was dominant, with a small amount of medium-amplitude δ wave	Normal	-	5 y	-	Normal
	4	Cerebral sulcus widened, ventricular dilation, corpus callosum thinning	Diffuse 1-4 Hz high amplitude mixed slow wave activity, mixed with a small amount of low amplitude fast wave activity, without dominant occipital rhythm	Absence	-	-	Normal	-
	5	Hydrocephalus	Generalized bihemispheric diffuse low-medium amplitude mixed slow wave activity	Absence	Bihemispheric multifocal sharp wave, sharp slow wave or irregular waveform asynchronous distribution or paroxysmal discharge, frontotemporal region and sleep period is obvious. Partial seizures	1 y	-	Severe developmental language delay, intellectual disability, motor developmental abnormality
	6	Ventricular dilation, bilateral extrafrontal space widened. Supratentorial hydrops	Diffuse medium-amplitude θ and δ waves in both hemispheres, mixed with slow wave activity, and no dominant occipital rhythm	Absence	Sharp wave, sharp slow wave, slow spike-wave, and slow wave discharged in the left middle and posterior temporal regions. Sporadic spike slow wave in the right Rolandic region during sleep	6 y	Normal	Developmental language delay, intellectual disability, motor developmental abnormality
	7	Severe hydrocephalus, cerebral white matter atrophy, interstitial edema, bilateral extracerebral space widened	Diffuse 1.5–2.5 Hz low-medium amplitude mixed slow wave activity in both hemispheres	Absence	Multiple focal spike waves, sharp waves, slow spike waves, and sharp slow waves were distributed asynchronously in bilateral hemispheres, especially in bilateral frontal, central, and anterior temporal regions. Intermittent voltage reduction in both hemispheres lasted for 1-5 s	5 y	Normal	Developmental language delay, intellectual disability, motor developmental abnormality
	8	Corpus callosum thinning	Hypsarrhythmia	Absence; hypsarrhythmia	Irregular spike slow waves, sharp waves, and slow waves were distributed asynchronously in the bilateral frontal pole, frontal and temporal regions during waking and sleeping periods. Spasm and atonic seizures	1 y	Died	Died
	9	Bilateral frontotemporal extracerebral space widened	Diffuse medium-amplitude θ and δ slow-wave activity was observed in both hemispheres without dominant occipital rhythm	Absence, increased slow wave	Low amplitude spike waves and slow spike waves are distributed in the frontal and temporal regions. Bihemispheric extensive spike slow wave is discharged briefly during sleep. Myoclonic seizures	3 y 6 m	Normal	Developmental language delay, intellectual disability, and motor developmental abnormality. Oral levetiracetam and topiramate were used for antiepileptic drugs
	10	Bilateral frontotemporal extracerebral space widened, cerebral sulcus widened.White matter change (especially high signal intensities on T2-weighted MRI in bilateral frontal lobes). Ventricular dilation, cerebral white matter atrophy, delayed myelination and corpus callosum thinning	Hypsarrhythmia	Absence; hypsarrhythmia	Irregular spike slow waves, sharp waves, and slow waves were distributed asynchronously in the frontal and temporal regions during waking and sleeping periods. Clusters of spasm seizures	3 y 2 m	Normal	Developmental language delay, intellectual disability, motor developmental abnormality
	11	Bilateral frontotemporal extracerebral space widened	Diffuse low-amplitude mixed slow-wave activity in both hemispheres	Absence	Irregular spikes and sharp waves distributed asynchronously on the bilateral posterior regions during the sleeping period	2 y	Normal	Movement developmental is normal, developmental language delay, intellectual disability
	12	Bilateral subdural effusion, extracerebral space widened. Patchy high signal intensities on T2-weighted and low signal on T1 weighted MRI in bilateral paraventricular white matter and basal ganglia regions	Diffuse low-amplitude mixed slow-wave activity in both hemispheres	Maturation was Absence	Slightly more multifocal sharp waves discharged mainly in the right occipitotemporal region in the waking and sleeping period,	4 y	Abnormal#	Movement developmental is normal, developmental language delay, intellectual disability
	13	Abnormal signals in the right basal ganglia region, paraventricular, and brain stem High signal intensities on T2-weighted and low signal on T1 weighted MRI in bilateral lateral ventricle anterior and posterior horn	Diffuse medium–high amplitude mixed slow wave activity in both hemispheres, no dominant occipital rhythm	Absence, slow wave increase	-	10 y	-	Motor developmental abnormality, developmental language delay, intellectual disability
	14	Bilateral frontoparietal temporal extracerebral space widened. Corpus callosum thinning	Diffuse medium amplitude mixed slow wave activity in both hemispheres	Normal	Spike slow waves and sharp slow waves discharged from the left occipital and temporal regions	5 y	-	Movement is developmental is normal, developmental language delay, intellectual disability
	15	Corpus callosum thinning. Bilateral centrum semiovale, anterior and posterior horn of bilateral ventricles demyelination. Hydrocephalus	Persistent diffuse medium-amplitude θ and δ slow-wave activity in both hemispheres	Absence	Multifocal Spike slow waves discharged in bilateral hemispheres	10 y	Normal	Developmental language delay, intellectual disability. Currently, oral levetiracetam is used for antiepileptic treatment
	16	Bilateral frontoparietal cerebral white matter atrophy, extracerebral space widened	Diffuse 2-4 Hz low amplitude mixed wave activity in both hemispheres	-	-	4 y	Abnormal#	Developmental language delay, intellectual disability, and motor developmental abnormality
**Isolated MMA**	17	Bilateral basal ganglia and cerebral peduncle cytotoxic edema	6-7 Hz low-medium amplitude θ activity in the bi-occipital region, generalized low voltage in both hemispheres	Normal	-	6 y	-	Developmental language delay, intellectual disability, and severe motor developmental abnormality
	18	Bilateral frontotemporal extracerebral space widened	Diffuse low-medium amplitude mixed slow wave activity in both hemispheres	Absence	-	5 y	Background rhythm was slowed	Normal

*N *number

*“*-*”*: No abnormality or seizure was detected, and the patient was lost to follow-up, “#”： examination in other hospitals (specific results cannot be provided). Brain rhythms can be divided into δ band, θ band, α band, β band and γ band. δ band:0.3–3.5 Hz, θ band:4–7 Hz, α band:8-13 Hz, β band:14-30 Hz, γ band:30-70 Hz

### Inclusion criteria

(1) Clinical symptoms of nervous system damage as the primary manifestation, such as consciousness disorders, poor feeding, developmental delay, convulsions, and intellectual disability. (2) The diagnostic criteria for MMA included serum propionylcarnitine (C3) and propionylcarnitine/acetylcarnitine (C2) (C3/C2) detected by tandem mass spectrometry. In addition, urine gas mass spectrometry detected elevated urine methylmalonic acid levels. Genetic testing confirmed the presence of the mutation. The secondary MMA of vitamin B12 deficiency was excluded [5].

### Auxiliary inspection

Blood routine, blood ammonia, blood lactic acid, blood homocysteine, liver and kidney function, brain MRI, and electroencephalogram (routine EEG, video EEG, and 24 h ambulatory electroencephalogram) were performed.

### Treatment and follow up

In the acute phase, all patients were intravenously administered levocarnitine (50–100 mg/kg, 1–2 times a day). After the symptoms were relieved, levocarnitine was given orally 100–300 mg/ (kg·d) (according to the clinical response and carnitine levels). Vitamin B12 1 mg intramuscular injection 2–3 times a week (depending on biochemical results) was administered to patients with a vitamin B12 responsive type (Rotexmedica, Germany). In our case, there was only one isolated MMA patient with an MUT gene mutation who did not respond to vitamin B12. This patient was administered a special formula (without isoleucine, methionine, threonine, and valine) and levocarnitine orally. During acute decompensation, patients may be intolerant to an enteral diet and may require intravenous infusion of glucose and electrolyte solutions to maintain water, electrolytes, acid–base balance, and energy support. Fluid replacement is typically performed using 10% glucose and electrolytes. Insulin was used to promote anabolism while maintaining normal glycemia [0.01 ~ 0.02 U/(kg·h)]. The rate and amount of fluid replacement were adjusted according to the patient's cardiac and renal functions. The amount of fluid was approximately 150 mL/kg/24 h and the duration did not exceed 24–48 h. One of the patients developed anemia with a red blood cell count of 1.8*10^12^/L, hemoglobin level of 61 g/L, and was infused with erythrocyte after filtering leukocyte (10–15 mL/kg/time, 3–5 mL/kg/h). One patient with mycoplasma infection and two with bacterial infection confirmed by blood bacterial culture were administered appropriate antibiotics. One patient with deep venous thrombosis of the lower extremity was administered a low-molecular-weight heparin sodium 0.3 mL subcutaneous injection. Simultaneously, it is necessary to control protein intake and blood ammonia levels during the acute phase. In addition, we administered betaine (200 mg/day, oral administration) and folinic acid (5–15 mg/day, oral administration) for long-term treatment. In all our cases, the blood ammonia level of all patients did not exceed 100 μmol/L, and it can be reduced to normal or close to normal levels through the treatment.

## Results

### General information and clinical features

In total, 18 patients were enrolled, including 15 males (83%) and 3 females (17%). Among them, 12 (67%) had an early onset and 4 (22%) had a late onset. There were 8 cases (44%) of convulsive symptoms, 16 (89%) of developmental delay, 6 (33%) of poor feeding, 8 (44%) of consciousness disturbance, 1 (5%) of dystonia, 3 (17%) of blood system changes (anemia and reduction of the three systems), 1 (5%) of deep venous thrombosis of the lower extremity, and 1 (5%) of acidosis (Table 1 and 2).

### Imaging data

**Fig. 1:**
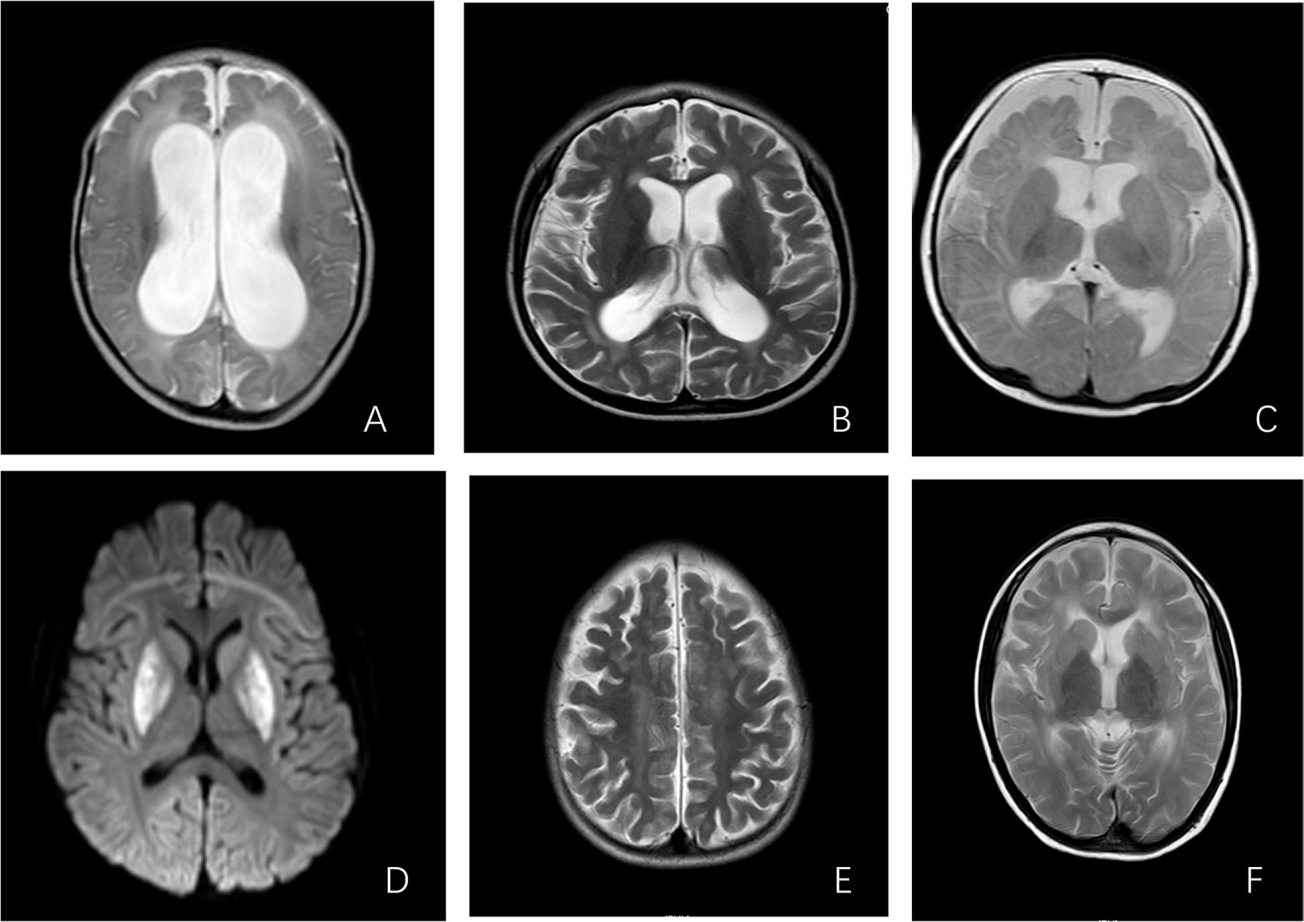
**A** Hydrocephalus, bilateral paraventricular interstitial edema (patient 2). **B** Corpus callosum thinning (patient 15). **C** Bilateral frontoparietal cerebral parenchymal atrophy, increased adjacent extracranial fluid (patient 16). **D** Bilateral putamen and globus pallidus involvement (patient 17). **E** Bilateral centrum semiovale dysmyelination (patient15). **F** Abnormal signals were observed in the nucleus caudatus and putamen (patient 13)

Mainly brain MRI. Examinations included T1, T2, Flair, and DWI, which included 6 cases (33%) of hydrocephalus, 9 (50%) of extracerebral space widened, 5 (27%) of corpus callosum thinning, 3 (17%) of ventricular dilation, 3 (17%) of abnormal signals in the brain parenchyma (frontal lobe, basal ganglia region, and brain stem), and 3 (17%) of abnormal signals in the lateral paraventricular. In addition, there were 3 cases (17%) of cerebral white matter atrophy and 1 case (5%) of cytotoxic edema in the basal ganglia and cerebral peduncle (Table 3, Fig. 1).

### EEG data

**Fig. 2:**
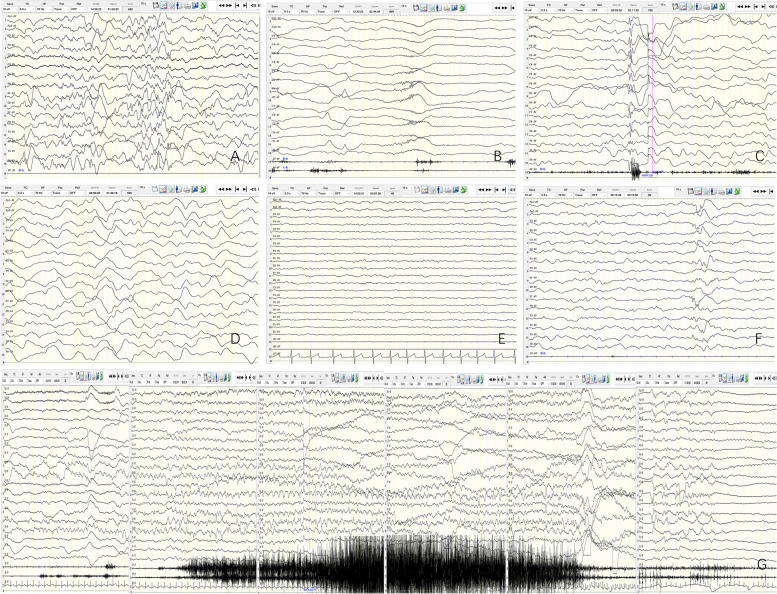
**A** Hypsarrhythmia (patient 8). **B** Spasm seizures (patient 10). **C** Myoclonic seizures (patient 9). **D** Diffuse 0.5–1.5 Hz high amplitude slow wave activity was observed in both hemispheres (patient 9). **E** Low-medium amplitude 4-6 Hz activity was observed in the bi-occipital region, generalized low voltage in both hemispheres (patient 17). **F** Sharp and slow waves were distributed in bilateral frontal region. (patient 7). **G** Focal seizure originated in the right occipital and posterior temporal region (patient 5)

EEG data showed 2 cases (11%) of hypsarrhythmia, 3 (17%) of voltage reduction, 12 (67%) of abnormal discharge, 13 (72%) of abnormal sleep physiological waves or abnormal sleep structure, 1 (5%) of immature (delayed) EEG development, and 8 (44%) of slow background. There were 2 cases (11%) of spasms, 1 (5%) of atonic seizures, and 1 (5%) of myoclonic seizures (Table 3, Fig. 2).

### Genetic testing

All 18 patients underwent genetic testing. MMACHC mutations accounted for 89% (16/18), all showing the cblC type MMA. MUT gene mutation accounted for 11% (2/18). The major mutation was c.609(exon4) G > A, accounting for 62.5% (10/16) (Table 1).

### Follow-up and prognosis

One patient was lost to follow-up, and one died. The remaining 16 patients were followed up at a mean age of  51.4 months (12–120 months). Blood homocysteine levels decreased in MMA patients after treatment, and there were no adverse drug reactions. In total, 87.5% (14/16) of children had varying developmental delays (intellectual disability, developmental language delay, and motor developmental abnormality). Development status temporarily nearly completely normal in two patients. EEG was re-examined in 11 cases, of which 8 were normal, and 3 were abnormal. In addition, 2 patients continued to take oral antiepileptic drugs at follow-up.

## Discussion

Methylmalonic acidemia is an autosomal recessive metabolic disease, and the most common genetic metabolic disease [12]. This is mainly due to metabolic defects caused by methyl malonyl coA mutase (MCM) or adenosylcobalamin (AdoCbl). The abnormal MCM leads to abnormal accumulation of metabolites such as methylmalonic acid, 3-hydroxybutyric acid, and methyl citrate, which damage the nervous system, liver, kidney, etc. [12].

The clinical manifestations of MMA are not specific but often occur with multiple organ involvement. Nevertheless, a few patterns of clinical presentations can be identified. Most patients exhibit developmental and cognitive impairment, feeding problems, neurological symptoms (seizures, movement disorders, abnormal muscle tone, ataxia, decreased consciousness, behavioral disorders, and mental disorders), visual impairment, hematological abnormalities, diseases of renal, cardiopulmonary, and gastrointestinal systems [13]. Using data from the E-HOD (homocystinuria and methylation defects) registry, Huemer et al. found that 89% of patients with cblC disease presented with early onset and 11% with late onset [14].

MMA can cause multisystem impairment and can occur over a wide age range, from newborns to adults. There are many subtypes of MMA, according to different gene mutation sites and biochemical tests. The pattern of clinical manifestations of cblC changes with age. For example, in the neonatal period, patients often experience neurological deterioration, manifested by lethargy, hypotonia, poor eating, epilepsy, and coma. Affected infants often present with stunted growth, anemia, and/or pancytopenia, as well as multisystem pathology, including renal and liver dysfunction and cardiomyopathy [15]. Older infants and young children often demonstrate acute encephalopathy and visual and cognitive impairment. Older children, adolescents, and adults may present with behavioral or mental disorders, cognitive impairment, peripheral neuropathy, and ataxia [16–18]. Ocular manifestations are rare in late-onset cblC, except for optic pallor [13]. In our study, most patients had central nervous system injuries. Approximately 44% of the patients had convulsions, 89% had developmental delays, 33% had poor feeding, 44% had disturbance of consciousness, 5% had dystonia, 17% had hematological abnormalities, 5% had acidosis, and 5% had deep venous thrombosis of the lower extremity. These symptoms gradually change with time; in childhood or adolescence, patients develop varying degrees of neurological symptoms such as developmental language delay, motor developmental abnormality, and intellectual disabilities.

Isolated MMA is associated with enzymatic subtypes *mut*^*0*^, *mut*^*–*^, *cblA*, *cblB*, and *cblD*-MMA [19]. The clinical manifestations of isolated MMA patients commonly present during the first weeks and months after birth are poor feeding, recurrent vomiting, and severe metabolic acidosis [20]. Moreover, infantile/non-B_12_-responsive isolated MMA patients always have infantile-onset lethargy, tachypnea, hypothermia, vomiting, and dehydration upon initiation of protein-containing feeds, which can rapidly progress to coma due to hyperammonemic encephalopathy when treatment is unavailable [19]. A study from Tsinghua University in China displayed a statistically significant difference in neurological findings between early- and late-onset isolated MMA, especially in developmental delay and movement disorders. Developmental delay was more common in early-onset patients, and movement disorders were more common in late-onset patients [12]. Of our two MUT patients, one was vitamin B12 non-responsive; however, surprisingly, his prognosis was relatively good, with good speech and movement, and he could attend school normally. We speculate that the reason for the good prognosis of this patient was that the brain parenchyma and electrophysiology of this child were not significantly affected, and this child received treatment early ( patient 18) [21]. Another 6-year-old MUT patient who was responsive to vitamin B12 had a poor prognosis and serious neurological sequelae. Currently, the child has grade III muscle strength in both lower limbs, grade III + muscle strength in both upper limbs, a positive Babinski sign, and dysarthria. Although the patient had late-onset MMA responsive to vitamin B12, the child initially had severe brain damage and EEG changes that were predictors of irreversible sequelae (patient 17).

MMA can affect brain development and lead to structural brain abnormalities. MRI can show white matter swelling and abnormal signals, corpus callosum thinning, hydrocephalus, and abnormal signals in the basal ganglia [22]. Myelin abnormalities, periventricular abnormalities, ventricular dilatation, brain atrophy, which can be significantly associated with developmental delay in children [23]. The pathogenesis of MMA combined with hydrocephalus remains unclear and may be linked to the direct neurotoxicity of toxic metabolites and oxidative stress response. For example, homocysteine can damage the vascular endothelium, stiffen arterial walls, decrease compliance, and affect the absorption of cerebrospinal fluid, leading to increased intracranial pressure and ventricular dilatation [24, 25]. It has been proposed that the correlation between central nervous system damage and neuropsychological developmental status in children with MMA can be evaluated based on MRI results to objectively evaluate the efficacy of standard treatment. They suggested that ventricular dilation is an important imaging feature in neuropsychological developmental disorders. In our study, the most common was extracerebral space widened, hydrocephalus, followed by corpus callosum thinning [23].

Electroencephalogram (EEG) examination is used to evaluate brain function, diagnose epilepsy, and identify episodic events. Moreover, EEG results may predict the outcomes of comatose patients; for example, non-reactive EEG, burst suppression mode, low voltage, and periodic epileptiform discharges indicate poor outcomes [26–29]. In our experiment, approximately half of the patients' EEG demonstrated background slowing, approximately 17% of the patients revealed low voltage, and approximately 72% showed changes in sleep structure, indicating changes in brain function and a decrease in normal physiological waves. In our study, two children had hypsarrhythmia and epileptic spasms, both of which were treated with ACTH and antiepileptic drugs; one died at approximately one year of age, and the other returned to normal EEG at approximately two years and three months. In the mechanism of central nervous system injury caused by MMA, mitochondrial injury, metabolic disorder, oxidative stress, and excitatory toxicity increase epilepsy occurrence [30]. Studies have revealed that nitric oxide (NO) has a protective effect on MMA, and the injection of L-arginine in the striatum can increase NO content in the striatum and reduce seizures induced by MMA [31]. The first symptom in 44% (8/18) of the patients in our study was convulsions, which was similar to that reported by Xiuwei Ma et al. [32]. During the follow-up, we found that 2 patients were still taking antiepileptic drugs. The electroencephalogram (EEG) of the 11 patients was reviewed, of which 8 had normal EEG and 3 had abnormal EEG. However, regardless of whether the EEG was normal, 87.5% of patients had developmental delays. It can be hypothesized that EEG can reveal whether MMA affects the brain physiology, disrupts neuronal functions and causes seizure activity in early period of the disease. However, long-term effects of MMA could not be anticipated with EEG.

The gene mutations involved in this study were mainly MMACHC and MUT. 89% of the cases were MMA with homocystinuria (cblC) type caused by MMACHC gene mutation, and 11% of cases were isolated MMA caused by MUT mutation. Consistent with most literature reports, the cblC type is the most common methylmalonic acid associated with homocysteinemia [33]. Studies on MMACHC gene mutations indicate a genotypic-phenotype correlation; for example, patients with c609G > A and 394 C > T tend to develop the late-onset disease, while those with c.331 C > T and c.271 dupA tend to appear in infancy [8, 34]. MUT gene mutation is the most common genotype of isolated MMA. In our case, the two isolated MMA were both MUT gene mutations, and there have been many reports about these two genetic variants [35–41].

Detailed guidelines for treating and managing MMA were developed by Baumgartner [1], Forny [1, 42], and Huemer et al. [13, 14, 43]. Among these, vitamin B12 therapy is one of the main drug treatments, but MMA patients have different responses to vitamin B12, which may be linked to different gene mutation types and sites. Patients with vitamin B12 response have a better prognosis. Those who did not respond to vitamin B12 had an early onset, and the first symptoms included lethargy, coma, and seizures [44]. However, despite aggressive treatment and improved metabolic levels, serious complications such as developmental delay can still occur [23]. Among these, nervous system injuries were the most significant. In this study, 87.5% of patients displayed mild or severe developmental delay during the follow-up process. Unfortunately, no objective developmental score test was conducted on the patients in our study because their families could not bear the economic and time costs of such examinations. In addition, the reference values and scoring items of the developmental score scales used by different hospitals differed, implying the absence of a uniform standard. Consequently, our study mainly obtained patient development from physical examinations and parent descriptions during follow-up. As for developmental language delay, some patients only speak simple words, overlapping words, and cannot understand complex sentences. Motor developmental abnormalities manifest as unstable walking, uncoordinated movement, and an inability to perform complex movements. Animal experiments have revealed that injection of methylmalonic acid into the lateral ventricle of mice can change the redox state, activate microglial cells, increase neural immunity, promote apoptosis, and alter several energy metabolic reactions in the brain (glucose, ATP, and oxidative metabolism), resulting in an insufficient energy supply to the brain. This proves that children with methylmalonic acid have brain dysfunction and cognitive changes [45, 46] and present with cognitive regression, mental confusion, and poor reaction ability [47]. Recent clinical data have demonstrated that peripheral blood inflammatory factors and oxidative stress products of patients with MMA have corresponding changes; these inflammatory factors destroy the blood–brain barrier, thus affecting cognition [48]. It has also been suggested that some patients with organic acidemia may present with bilateral basal ganglia necrosis due to excitatory toxicity caused by metabolic disorders [49]. Recent studies have demonstrated that post-translational modifications of some enzymes or proteins can cause metabolic disorders, thereby affecting brain function [50].

## Conclusion

The clinical manifestations of MMA vary, and its diagnosis based on the clinical symptoms is difficult. The possibility of an inherited metabolic disease should be considered when unexplained neurological or other systemic abnormalities are present. Biochemical examination, plasma acylcarnitine, urine organic acids, and genetic tests confirmed the diagnosis. MMA has a high mortality rate and a poor prognosis. Therefore, early diagnosis and treatment are required to reduce irreversible complications.

## Data Availability

The datasets generated or analyzed during this study are available from the first author upon reasonable request. First author: Yujun Yuan.

## References

[1] Forny P, Hörster F, Ballhausen D, Chakrapani A, Chapman KA, Dionisi-Vici C (2021). Guidelines for the diagnosis and management of methylmalonic acidaemia and propionic acidaemia: First revision. J Inherit Metab Dis.

[2] Tu WJ (2011). Methylmalonic acidemia in mainland China. Ann Nutr Metab.

[3] Qiliang L, Wenqi S, Quan W, Xinying Y, Jiuwei L, Qiang S (2015). Predictors of survival in children with methymalonic acidemia with homocystinuria in Beijing, China: a prospective cohort study. Indian Pediatr.

[4] Zhou X, Cui Y, Han J (2018). Methylmalonic acidemia: Current status and research priorities. Intractable Rare Dis Res.

[5] Han B, Cao Z, Tian L, Zou H, Yang L, Zhu W (2016). Clinical presentation, gene analysis and outcomes in young patients with early-treated combined methylmalonic acidemia and homocysteinemia (cblC type) in Shandong province. China Brain Dev.

[6] Weisfeld-Adams JD, Bender HA, Miley-Åkerstedt A, Frempong T, Schrager NL, Patel K (2013). Neurologic and neurodevelopmental phenotypes in young children with early-treated combined methylmalonic acidemia and homocystinuria, cobalamin C type. Mol Genet Metab.

[7] Chen T, Gao Y, Zhang S, Wang Y, Sui C, Yang L (2023). Methylmalonic acidemia: Neurodevelopment and neuroimaging. Front Neurosci.

[8] Lerner-Ellis JP, Anastasio N, Liu J, Coelho D, Suormala T, Stucki M (2009). Spectrum of mutations in MMACHC, allelic expression, and evidence for genotype-phenotype correlations. Hum Mutat.

[9] Chen Z, Dong H, Liu Y, He R, Song J, Jin Y (2022). Late-onset cblC deficiency around puberty: a retrospective study of the clinical characteristics, diagnosis, and treatment. Orphanet J Rare Dis.

[10] Tsai ACH, Morel CF, Scharer G, Yang M, Lerner-Ellis JP, Rosenblatt DS (2007). Late-onset combined homocystinuria and methylmalonic aciduria (cblC) and neuropsychiatric disturbance. Am J Med Genet A.

[11] Martinez Alvarez L, Jameson E, Parry NRA, Lloyd C, Ashworth JL (2016). Optic neuropathy in methylmalonic acidemia and propionic acidemia. Br J Ophthalmol.

[12] Kang L, Liu Y, Shen M, Liu Y, He R, Song J (2020). A study on a cohort of 301 Chinese patients with isolated methylmalonic acidemia. J Inherit Metab Dis.

[13] Guidelines for diagnosis and management of the cobalamin‐related remethylation disorders cblC, cblD, cblE, cblF, cblG, cblJ and MTHFR deficiency - Huemer - 2017 - Journal of Inherited Metabolic Disease - Wiley Online Library. Available from: https://onlinelibrary.wiley.com/doi/10.1007/s10545-016-9991-4. Cited 2023 Dec 4. 10.1007/s10545-016-9991-4 PMC5203859 27905001

[14] Huemer M, Diodato D, Martinelli D, Olivieri G, Blom H, Gleich F (2019). Phenotype, treatment practice and outcome in the cobalamin-dependent remethylation disorders and MTHFR deficiency: Data from the E-HOD registry. J of Inher Metab Disea.

[15] Weisfeld-Adams JD, Morrissey MA, Kirmse BM, Salveson BR, Wasserstein MP, McGuire PJ (2010). Newborn screening and early biochemical follow-up in combined methylmalonic aciduria and homocystinuria, cblC type, and utility of methionine as a secondary screening analyte. Mol Genet Metab.

[16] Bodamer OA, Rosenblatt DS, Appel SH, Beaudet AL (2001). Adult-onset combined methylmalonic aciduria and homocystinuria (cblC). Neurology.

[17] Van Hove JLK, Van Damme-Lombaerts R, Grünewald S, Peters H, Van Damme B, Fryns JP (2002). Cobalamin disorder Cbl-C presenting with late-onset thrombotic microangiopathy. Am J Med Genet.

[18] Thauvin-Robinet C, Roze E, Couvreur G, Horellou MH, Sedel F, Grabli D (2008). The adolescent and adult form of cobalamin C disease: clinical and molecular spectrum. J Neurol Neurosurg Psychiatry.

[19] Manoli I, Sloan JL, Venditti CP. Isolated Methylmalonic Acidemia. In: Adam MP, Everman DB, Mirzaa GM, Pagon RA, Wallace SE, Bean LJ, et al., editors. GeneReviews®. Seattle (WA): University of Washington, Seattle; 1993. Available from: http://www.ncbi.nlm.nih.gov/books/NBK1231/. Cited 2022 Nov 28.

[20] Zhou W, Li H, Wang C, Wang X, Gu M (2018). Newborn Screening for Methylmalonic Acidemia in a Chinese Population: Molecular Genetic Confirmation and Genotype Phenotype Correlations. Front Genet.

[21] He R, Mo R, Shen M, Kang L, Song J, Liu Y (2020). Variable phenotypes and outcomes associated with the MMACHC c.609G>A homologous mutation: long term follow-up in a large cohort of cases. Orphanet J Rare Dis..

[22] Longo D, Fariello G, Dionisi-Vici C, Cannatà V, Boenzi S, Genovese E (2005). MRI and 1H-MRS Findings in Early-Onset Cobalamin C/D Defect. Neuropediatrics.

[23] Yang L, Guo B, Li X, Liu X, Wei X, Guo L (2020). Brain MRI features of methylmalonic acidemia in children: the relationship between neuropsychological scores and MRI findings. Sci Rep.

[24] Chen Z, Karaplis AC, Ackerman SL, Pogribny IP, Melnyk S, Lussier-Cacan S (2001). Mice deficient in methylenetetrahydrofolate reductase exhibit hyperhomocysteinemia and decreased methylation capacity, with neuropathology and aortic lipid deposition. Hum Mol Genet.

[25] Sachdev PS (2005). Homocysteine and brain atrophy. Prog Neuropsychopharmacol Biol Psychiatry.

[26] Claassen J, Mayer SA (2002). Continuous electroencephalographic monitoring in neurocritical care. Curr Neurol Neurosci Rep.

[27] Admiraal MM, van Rootselaar AF, Hofmeijer J, Hoedemaekers CWE, van Kaam CR, Keijzer HM (2019). Electroencephalographic reactivity as predictor of neurological outcome in postanoxic coma: A multicenter prospective cohort study. Ann Neurol.

[28] You W, Tang Q, Wu X, Feng J, Mao Q, Gao G (2018). Amplitude-Integrated Electroencephalography Predicts Outcome in Patients with Coma After Acute Brain Injury. Neurosci Bull.

[29] Azabou E, Navarro V, Kubis N, Gavaret M, Heming N, Cariou A (2018). Value and mechanisms of EEG reactivity in the prognosis of patients with impaired consciousness: a systematic review. Crit Care.

[30] Rho JM, Boison D (2022). The metabolic basis of epilepsy. Nat Rev Neurol.

[31] Royes LFF, Fighera MR, Furian AF, Oliveira MS, Fiorenza NG, Petry JC (2007). The role of nitric oxide on the convulsive behavior and oxidative stress induced by methylmalonate: an electroencephalographic and neurochemical study. Epilepsy Res.

[32] Ma X, Zhang Y, Yang Y, Liu X, Yang Z, Bao X (2011). Epilepsy in children with methylmalonic acidemia: electroclinical features and prognosis. Brain Dev.

[33] Brox-Torrecilla N, Arhip L, Miguélez-González M, Castellano-Gasch S, Contreras-Chicote A, Rodríguez-Ferrero ML (2021). Late-onset methylmalonic acidemia and homocysteinemia. Nutr Hosp.

[34] Wang F, Han L, Yang Y, Gu X, Ye J, Qiu W (2010). Clinical, biochemical, and molecular analysis of combined methylmalonic acidemia and hyperhomocysteinemia (cblC type) in China. J Inherit Metab Dis.

[35] Morel c’c, Lerner-Ellis JP, Rosenblatt DS (2006). Combined methylmalonic aciduria and homocystinuria (cblC): Phenotype–genotype correlations and ethnic-specific observations. Mol Genet Metabol..

[36] He RX, Dong H, Zhang HW, Zhang Y, Kang LL, Li H (2021). Clinical and genetic studies on 76 patients with hydrocephalus caused by methylmalonic acidemia combined with homocysteinuria. Zhonghua Er Ke Za Zhi.

[37] Xiong H, Deng W, Guo L, Shi C, Xiao X, Hao H (2019). Clinical and variant analysis of 15 patients with methylmalonic acidemia. Zhonghua Yi Xue Yi Chuan Xue Za Zhi.

[38] He R, Mo R, Zhang Y, Shen M, Kang L, Chen Z (2022). Factors affecting phenotypes in the patients with MMACHC gene c.609G>A homozygous variant cblC type methylmalonic acidemia combined with homocysteinuria. Zhonghua Yi Xue Yi Chuan Xue Za Zhi..

[39] Wang X, Sun X, Hao S, Liu F, Zhang Q, Zheng L (2022). Genetic analysis of 21 cases of methylmalonic acidemia. Zhonghua Yi Xue Yi Chuan Xue Za Zhi.

[40] Panigrahi I, Bhunwal S, Varma H, Singh S (2017). Methylmalonic Acidemia with Novel MUT Gene Mutations. Case Rep Genet.

[41] Kang LL, Liu YP, Shen M, Chen ZH, Song JQ, He RX (2020). The phenotypes and genotypes in 314 patients with isolated methylmalonic acidemia. Zhonghua Er Ke Za Zhi.

[42] Forny P, Froese DS, Suormala T, Yue WW, Baumgartner MR (2014). Functional characterization and categorization of missense mutations that cause methylmalonyl-CoA mutase (MUT) deficiency. Hum Mutat.

[43] Huemer M, Simma B, Fowler B, Suormala T, Bodamer OA, Sass JO (2005). Prenatal and postnatal treatment in cobalamin C defect. J Pediatr.

[44] Yu Y, Shuai R, Liang L, Qiu W, Shen L, Wu S (2021). Different mutations in the MMUT gene are associated with the effect of vitamin B12 in a cohort of 266 Chinese patients with mut-type methylmalonic acidemia: A retrospective study. Mol Genet Genomic Med.

[45] Gabbi P, Ribeiro LR, Jessié Martins G, Cardoso AS, Haupental F, Rodrigues FS (2017). Methylmalonate Induces Inflammatory and Apoptotic Potential: A Link to Glial Activation and Neurological Dysfunction. J Neuropathol Exp Neurol.

[46] Wajner M, Coelho JC (1997). Neurological dysfunction in methylmalonic acidaemia is probably related to the inhibitory effect of methylmalonate on brain energy production. J Inherit Metab Dis.

[47] Dienel GA. Brain Glucose Metabolism: Integration of Energetics with Function. Physiological Reviews. 2018. Available from: https://journals.physiology.org/doi/10.1152/physrev.00062.2017. Cited 2023 Feb 10. 10.1152/physrev.00062.2017 30565508

[48] Li Q, Jin H, Liu Y, Rong Y, Yang T, Nie X (2021). Determination of Cytokines and Oxidative Stress Biomarkers in Cognitive Impairment Induced by Methylmalonic Acidemia. NeuroImmunoModulation.

[49] Ludolph AC, Riepe M, Ullrich K (1993). Excitotoxicity, energy metabolism and neurodegeneration. J Inherit Metab Dis.

[50] Head PE, Myung S, Chen Y, Schneller JL, Wang C, Duncan N (2022). Aberrant methylmalonylation underlies methylmalonic acidemia and is attenuated by an engineered sirtuin. Sci Transl Med..

